# Fatal Breakthrough Candidemia in an Immunocompromised Patient in Kuwait Due to *Candida auris* Exhibiting Reduced Susceptibility to Echinocandins and Carrying a Novel Mutation in Hotspot-1 of *FKS1*

**DOI:** 10.3390/jof8030267

**Published:** 2022-03-06

**Authors:** Inaam Al-Obaid, Mohammad Asadzadeh, Suhail Ahmad, Khaled Alobaid, Wadha Alfouzan, Ritu Bafna, Maha Emara, Leena Joseph

**Affiliations:** 1 Department of Microbiology, Al-Sabah Hospital, Shuwaikh 70031, Kuwait; inaob218@hotmail.com (I.A.-O.); drritubafna@gmail.com (R.B.); maha.memara@gmail.com (M.E.); 2 Department of Microbiology, Faculty of Medicine, Kuwait University, Safat 13110, Kuwait; mohammad.assadzadeh@ku.edu.kw (M.A.); alfouzan.w@ku.edu.kw (W.A.); leena.anniejoseph@ku.edu.kw (L.J.); 3 Department of Microbiology, Mubarak Al-Kabeer Hospital, Jabriya 46300, Kuwait; khaled22m@live.com

**Keywords:** *C. auris*, breakthrough candidemia, echinocandin resistance, hotspot-1 of *FKS1*, S639T mutation, immunocompromised patient

## Abstract

*Candida auris* is an emerging yeast pathogen that has recently caused major outbreaks in healthcare facilities worldwide. Clinical *C. auris* isolates are usually resistant to fluconazole and readily develop resistance to echinocandins and amphotericin B (AMB) during treatment. We describe here an interesting case of *C. auris* infection in an immunocompromised patient who had previously received AMB and caspofungin treatment. Subsequently, *C. auris* was isolated from tracheal (tracheostomy) secretions and twice from urine and all three isolates were susceptible to AMB and micafungin. The patient received a combination therapy with AMB and caspofungin. Although the *C. auris* was cleared from the urine, the patient subsequently developed breakthrough candidemia and the bloodstream isolate exhibited a reduced susceptibility to micafungin and also showed the presence of a novel (S639T) mutation in hotspot-1 of *FKS1*. Interestingly, *C. auris* from the tracheal (tracheostomy) secretions recovered one and four days later exhibited a reduced susceptibility to micafungin and S639Y and S639T mutations in hotspot-1 of *FKS1*, respectively. Although the treatment was changed to voriconazole, the patient expired. Our case highlights a novel *FKS1* mutation and the problems clinicians are facing to treat invasive *C. auris* infections due to inherent or developing resistance to multiple antifungal drugs and limited antifungal armamentarium.

## 1. Background

The incidence of invasive fungal infections (IFIs) is continuously increasing throughout the world due to an increasing population of immunocompromised patients, and IFIs are associated with high mortality rates [1,2]. *Candida* spp. are a common cause of IFIs and candidemia represents nearly 75% of all invasive *Candida* infections [3,4,5]; however, blood culture positivity for deep-seated candidiasis is usually less than 50%, suggesting that many cases are not detected by routine laboratory diagnostic methods [6,7,8]. Due to a lower culture positivity and non-specific signs and symptoms of the disease, patients with suspected invasive candidiasis are typically treated empirically with antifungal drugs [4,9]; however, patients receiving systemic antifungals also become susceptible to breakthrough infections with drug-resistant fungal pathogens [2,10]. Several reports have described breakthrough candidemia cases with drug-resistant/multidrug-resistant *Candida* among hospitalized patients receiving antifungal drugs as prophylaxis or empiric therapy, particularly among patients with hematological malignancies or other conditions compromising their immune defenses [11,12,13,14,15,16,17,18].

The epidemiology of invasive candidiasis has changed dramatically in the last two decades due to changes in clinical practice and the increasing use of fluconazole and other antifungal drugs for prophylaxis and/or treatment of at-risk patients, particularly in intensive care unit (ICU) settings [9,19,20]. Since *C. albicans* is usually highly susceptible to antifungal drugs including fluconazole, this has led to the emergence of drug-resistant non-*albicans Candida* spp. as a significant cause of invasive candidiasis in many geographical locations [5,21,22,23,24,25,26,27]. *Candida auris* is a recently described, often multidrug-resistant, pathogenic yeast that has caused nosocomial invasive infections and outbreaks in more than 47 countries worldwide [28,29]. This novel yeast is easily transmitted among hospitalized patients as it colonizes the skin/nares, is shed into the environment contaminating hospital surfaces and equipment and can survive even under harsh conditions [28,29]. Like other *Candida* spp., invasive *C. auris* infections also occur more easily among colonized patients who are critically ill with prolonged hospitalization and have several comorbidities, particularly diabetes mellitus, respiratory disease or chronic kidney disease as well as indwelling catheters, mechanical ventilation and/or admission in the ICU [18,27,30,31,32,33,34,35]. A systematic review and meta-analysis have reported an over-all mortality rate of nearly 39% among *C. auris*-infected patients [36].

The aim of this study is to describe a fatal case of breakthrough *C. auris* candidemia in a lung transplant patient who was colonized with *C. auris* and was receiving combination therapy with AMB and caspofungin. We also discuss the role of different mutations which have been described so far in hotspot-1 and hotspot-2 of *FKS1* and confer a reduced susceptibility to echinocandins in *C. auris*.

## 2. Case Report

**Figure 1:**
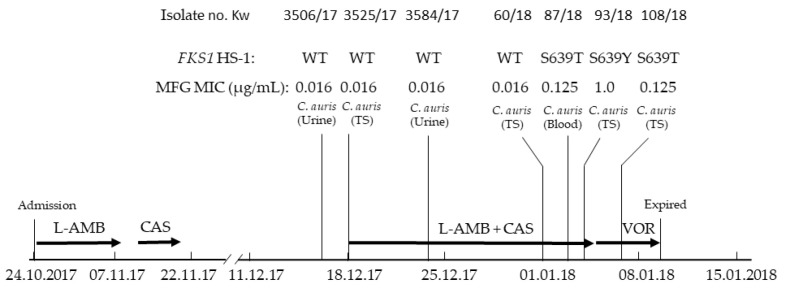
Time-line for the isolation of *C. auris*, antifungal treatment given to the patient and outcome. L-AMB, liposomal amphotericin B; CAS, caspofungin; MFG, micafungin; VOR, voriconazole; TS, tracheal secretion; *FKS1* HS-1, hotspot-1 region of *FKS1* gene; WT, wild-type.

The patient, a 32-year old bilateral lung transplant recipient in 2014 with type 1 diabetes mellitus, an inferior vena cava filter for previous deep venous thrombosis, chronic transplant rejection and respiratory failure, was admitted in the ICU of our hospital on 24 October 2017 (Day 1). On admission, she was severely hypotensive (70/30 mmHg), hypothermic (35.5 °C) and her Glasgow Coma Scale was 9/15. She was placed on mechanical ventilation, through tracheostomy (FIO_2_ was 50%), and was also on inotropes. Laboratory investigations revealed a peripheral leukocyte count of 7.4 × 10^9^/L; serum creatinine, 54 µmol/L and C-reactive protein (CRP), 129.3 mg/L. She was treated empirically with meropenem (1 g q8h, 16 days), amikacin (500 mg q12h, 3 days), colistin nebulization (2MU q8h), teicoplanin (200 mg q12h, 10 days), ganciclovir (150 mg q12h, 9 days) and liposomal amphotericin B (L-AMB) (200 mg q24h, 16 days) (Figure 1). Her cultures revealed no growth. One week later she improved clinically and noradrenaline was tapered down. On Day 20, she developed diarrhea with rectal bleeding and she was hypotensive. There was no peripheral leukocytosis, but the CRP was raised (711.5 mg/L). Blood and urine cultures were negative. The tracheostomy secretion yielded scanty growth of *Candida krusei* and caspofungin (70 mg loading dose and then 50 mg q24h) was given for seven days. A colonoscopy was performed and revealed colitis while a colon biopsy showed cytomegalovirus infection, hence, ganciclovir (150 mg q12h) was added, and she improved clinically.

On Day 48, the patient became hemodynamically unstable and was started on 0.1 µg/kg/min noradrenaline. She was afebrile and there was no peripheral leukocytosis, but the CRP was raised up to 252 mg/L. On Day 49, the blood culture grew coagulase-negative staphylococci and teicoplanin (200 mg q12h) was started. After an initial improvement, the patient deteriorated and required inotropic support. On Day 54, the urine culture yielded yeast growth (Isolate Kw3506/17) which was initially identified as *C. haemulonii* by Vitek2 but was later confirmed as *C. auris* by molecular methods. On Day 56, the tracheal (tracheostomy) aspirate also yielded *C. auris* (Isolate Kw3525/17) (Figure 1). The patient was started on a combination of L-AMB (300 mg q24h) and caspofungin (70 mg loading dose and then 50 mg q24h). The patient did not show much clinical improvement and on Day 62, *C. auris* (Isolate Kw3584/17) (Figure 1) was again cultured from urine but the growth was scanty. Fourteen days into the combination therapy with L-AMB and caspofungin, heavy growth of *C. auris* (Isolate Kw60/18) was obtained from the tracheal secretion on Day 70, but the urine culture did not yield *C. auris*. Two days later (Day 72), the patient developed candidemia as the blood drawn from the central line yielded *C. auris* (Isolate Kw87/18) (Figure 1). On Day 73, *C. auris* (Isolate Kw93/18) was isolated again from the tracheal (tracheostomy) aspirate. Following the identification of bloodstream yeast isolate as *C. auris*, the combination therapy with L-AMB and caspofungin was stopped and the patient was started on voriconazole. On Day 76, the tracheal (tracheostomy) aspirate again yielded *C. auris* (Isolate Kw108/18) and three days later (Day 79), the patient expired (Figure 1).

### Isolation, Molecular Characterization and Antifungal Susceptibility Testing

The clinical samples were processed according to standard laboratory procedures described previously [37]. The blood specimens were processed by an automated BACTEC 9240 system (Becton Dickinson, Sparks, MD, USA) and yeast isolates were provisionally identified by the Vitek2 yeast identification system (bioMerieux, Marcy I’Etoile, France) as described previously [38]. The isolates were sent to the Mycology Reference Laboratory (MRL) for molecular identification and antifungal drug susceptibility testing. Genomic DNA was prepared from each isolate by the rapid boiling method [39]. Species-specific identification was achieved by PCR amplification of the rDNA by using *C. auris*-specific primers and by PCR amplification of the rDNA followed by DNA sequencing using pan-fungal primers, as described previously [37,40]. In vitro susceptibility to antifungal drugs was obtained against eight (fluconazole, voriconazole, itraconazole, posaconazole, anidulafungin, micafungin, amphotericin B and 5-flucytosine) antifungal drugs by the commercial MICRONAUT-AM antifungal susceptibility testing (AST) panel for yeasts (Merlin Diagnostica GmbH, Bornheim, Germany). The procedure was performed by following the instructions supplied with the kit and as described previously [34]. Reference strains of *Candida krusei* (ATCC 6258) and *Candida parapsilosis* (ATCC 22019) were processed simultaneously during the AST to ensure quality control. The minimum inhibitory concentration (MIC) values were determined after 24 h of incubation at 35 °C as described previously [34]. Since there are no *C. auris*-specific susceptibility breakpoints, tentative breakpoints of ≥32 µg/mL for the fluconazole, ≥2 µg/mL for voriconazole, ≥2 µg/mL for itraconazole, ≥2 µg/mL for posaconazole, ≥4 µg/mL for anidulafungin, ≥4 µg/mL for micafungin and ≥2 µg/mL for the amphotericin B, as proposed by the Centers for Disease Control and Prevention (CDC) of the USA and expert opinion were used [41,42,43,44].

Fluconazole resistance-conferring mutations in *ERG11* were detected by PCR-sequencing, as described previously [45]. The mutations conferring resistance to micafungin in *C. auris* strains are usually located in the hotspot-1 or hotspot-2 regions of the *FKS1* gene [18,28,46]. Mutations in the hotspot-1 and hotspot-2 regions of *FKS1* were detected by PCR-sequencing, as described previously [18,45].

A total of seven *C. auris* were isolated from different clinical specimens during the 23 day (16 December 2017 to 7 January 2018) period. All the isolates were identified as *C. auris* by species-specific PCR amplification, and their ITS region of rDNA sequences were identical and showed 100% identity with *C. auris* strains isolated previously from Kuwait and India. The AST data showed that all seven isolates were uniformly resistant to fluconazole but appeared susceptible to other triazoles (Table 1). Sequence analyses of the hotspot region of *ERG11* identified a fluconazole resistance-conferring K143R mutation in all seven isolates (Table 1). All seven isolates appeared susceptible to AMB and 5-flucytosine; however, their susceptibility to echinocandins varied. While the two urine isolates and the first two isolates from the tracheal (tracheostomy) secretions were susceptible to micafungin (and anidulafungin), the bloodstream and the two subsequent isolates from tracheal (tracheostomy) secretions showed a reduced susceptibility to micafungin (and anidulafungin) (Table 1). Consistent with the AST data for the two echinocandins, the two (Kw3506/17 and Kw3584/17) urine isolates and the first two (Kw3525/17 and Kw60/18) isolates from tracheal secretions contained a wild-type sequence of hotspot-1 of *FKS1*; however, the bloodstream isolate (Kw87/18) showing a reduced susceptibility to micafungin (MIC 0.125 µg/mL), contained a S639T mutation in hotspot-1 of *FKS1*. Surprisingly, the *C. auris* (Kw93/18) isolated from tracheal secretions one day later and exhibiting a reduced susceptibility to micafungin (MIC 1 µg/mL), contained a S639Y mutation in hotspot-1 of *FKS1*; however, another *C. auris* (Kw108/18) isolated subsequently from the tracheal secretions and exhibiting a reduced susceptibility to micafungin (MI 0.125 µg/mL) contained a S639T mutation in the hotspot-1 of *FKS1*. All three latter isolates contained a wild-type sequence of the hotspot-2 region of *FKS1* (Table 1).

## 3. Comments

The emergence of breakthrough IFIs, particularly due to drug-resistant strains of *Candida* and other yeast species, is now regarded as a significant threat to immunocompromised patients in ICU settings receiving systemic antifungal drugs as prophylaxis [2,10,30]. In this study, we described a fatal case of breakthrough candidemia due to *C. auris* in an immunocompromised patient who had previously received more than one course of antifungal prophylaxis or empiric treatment. The patient was colonized initially with *C. auris* in the urinary tract and subsequently also in the respiratory tract. Interestingly, infection from the urinary tract was successfully cleared due to combination therapy with liposomal AMB and caspofungin. Candiduria due to *C. auris* was also successfully eradicated by therapy with liposomal AMB in another recent study in an immunocompromised patient [18]. Since AMB is excreted through the kidneys, these findings suggest that treatment with AMB is an attractive alternative to clear urinary tract infection due to *C. auris* in hospitalized patients.

Despite clearance of the *C. auris* infection from the urinary tract, breakthrough candidemia due to *C. auris* developed while the patient was still receiving combination therapy with liposomal AMB and caspofungin. The bloodstream isolate was resistant to the fluconazole but exhibited a reduced susceptibility (MIC 0.125 µg/mL) to micafungin while the four colonizing strains obtained previously were resistant to fluconazole but were highly susceptible (MIC 0.016 µg/mL) to micafungin. Furthermore, unlike the four colonizing strains which contained the wild-type sequence, the bloodstream isolate displayed a novel mutation (S639T) in hotspot-1 of *FKS1* which has not been described previously among echinocandin-resistant *C. auris* [18,34,43,45,46,47,48]. Although the *C. auris* isolate recovered from the tracheal aspirate one day later also exhibited a reduced susceptibility (MIC 1 µg/mL) to micafungin, it contained an S639Y mutation in hotspot-1 of *FKS1* while another *C. auris* isolate from the tracheal aspirate recovered three days later was similar to the bloodstream isolate in terms of both susceptibility to micafungin (MIC 0.125 µg/mL) and the *FKS1* sequence (S639T mutation in hotspot-1 of *FKS1*). All seven *C. auris* isolates contained an identical ITS region of rDNA sequences and the fluconazole resistance-conferring K143R mutation in *ERG11*; however, it could not be ascertained whether they were clonally related as detailed high-resolution fingerprinting by short tandem repeat typing or by whole genome sequencing was not carried out.

It is reasonable to assume that the reduced susceptibility to echinocandins first developed in the *C. auris* colonizing the tracheal region of the respiratory tract in our patient which subsequently translocated to the bloodstream, as was also shown in a case of breakthrough candidemia in a recent study by Asadzadeh et al. [18]. *C. auris* exhibiting a reduced susceptibility to echinocandins and carrying a novel (R1354H) mutation in hotspot-2 of *FKS1*, was isolated from the bloodstream of an immunocompromised patient who was also colonized in the respiratory tract with this yeast carrying the same rare mutation in hotspot-2 of *FKS1* [18]. The isolation of *C. auris*, however, with a reduced susceptibility to micafungin and carrying the S639Y mutation from a tracheal (tracheostomy) secretion before the isolation of another isolate carrying the S639T mutation—similar to the bloodstream isolate—is intriguing but could be explained as follows. Since the *C. auris* from the bloodstream and other anatomic sites were subcultured on Sabouraud dextrose agar and single colonies were selected for further studies and the storage of cultures, it is probable that both *C. auris* with S639Y and with S639T mutations evolved simultaneously from echinocandin-susceptible *C. auris* in the tracheal region of the respiratory tract, but only the isolate with the S639Y mutation was detected by chance in the culture grown on 4 January 2018. Meanwhile, the *C. auris* with the S639Y mutation was cleared, likely due to its reduced fitness while the *C. auris* with the S639T mutation proliferated as it probably had little, if any, fitness issue. This is similar to the development of drug resistance in another pathogen, *Mycobacterium tuberculosis*. The resistance of *M. tuberculosis* to isoniazid is caused mainly by mutations in the *katG* gene and a S315T mutation in *katG* causes high-level resistance to isoniazid and minimal effect on fitness, which eventually leads to multidrug resistance when the *M. tuberculosis* additionally develops a resistance to rifampicin [49,50]. Although other (S315N, S315I, S315R and S315G) mutations have also been described in a minority of isoniazid-resistant *M. tuberculosis* strains, isolates with the S315Y mutation have not been detected so far [51].

The present case also shows that the MIC of the *C auris* isolate to echinocandins is not a good predictor of outcome as the patient developed breakthrough candidemia due to the *C. auris* while on therapy with caspofungin, even though the isolate showed only a moderate increase in the MIC value for micafungin and the patient died soon after the diagnosis of an invasive infection. A similar fatal outcome was also recently reported in a patient who experienced breakthrough candidemia with *C. auris* [18]. In their study, the patient experienced breakthrough candidemia while on therapy with caspofungin even though the MIC of the *C. auris* isolate for micafungin and carrying a R1354H mutation in *FKS1* was only 0.125 µg/mL [18]. Another recent study has also concluded that the *FKS1* genotype rather than the MIC of the *C. auris* for echinocandins is a more accurate predictor of an in vivo response to treatment in the mouse model of disseminated infection [46].

Mutations in the hotspot-1 and hotpot-2 regions of *FKS1* usually confer a reduced susceptibility to echinocandins in *C. auris* [18,43,45,46,48,51,52,53]. Until recently, only a few non-synonymous mutations at codon 639 within the hotspot-1 of *FKS1* were described among echinocandin-resistant clinical *C. auris* isolates in different studies; however, more recently, several novel mutations have been described not only at other codon positions within hotspot-1 but also in hotspot-2 of *FKS1* and are summarized in Table 2. Three codon positions (F635, S639 and D642) within hotspot-1 and a single codon position (R1354) within hotspot-2 of *FKS1 were* mutated in the *C. auris* isolates exhibiting a reduced susceptibility to echinocandins. It is also apparent from the data reported in Table 2 that the *C. auris* strains with an identical *FKS1* mutation exhibited variable susceptibility to echinocandins (as illustrated by the differences in their MIC values) likely due to differences in their genetic background, as global *C. auris* strains belong to one of five Clades which differ in thousands of single nucleotide polymorphisms and exhibit considerable differences in their intrinsic susceptibility to antifungal drugs [44,54]. Additionally, many of the *C. auris* strains described in various studies may have been isolated from immunocompromised patients who had likely been previously exposed to antifungal drugs on multiple occasions as a prophylaxis or empiric treatment and, therefore, may have accumulated mutations in other genes that alter permeability barriers resulting in differences in susceptibility to different antifungal drugs [18,53,55,56]. Another interesting point that has been highlighted recently is the variable in vivo response to treatment with antifungal drugs among *C. auris* strains carrying different mutations in hotspot-1 or hotspot-2 of *FKS1* [46]. These findings strongly suggest that *C. auris* with different *FKS1* mutations may also differ with respect to their survival in the hostile environment within the human host, i.e., they exhibit variable fitness defects.

## 4. Conclusions

We presented a unique case of fatal breakthrough candidemia due to *C. auris* in an immunocompromised patient while receiving combination therapy with liposomal amphotericin B and caspofungin. The *C. auris* from the bloodstream as well as from tracheal secretions exhibited a reduced susceptibility to micafungin and showed the presence of a novel (S639T) mutation in hotspot-1 of the *FKS1* gene which has not been described previously. It is proposed that among all non-synonymous mutations detected so far at codon S639 within the hotspot-1 of *FKS1*, isolates with a S639T mutation may be least affected by the fitness cost imposed by the genetic alteration.

The DNA sequencing data reported in this study have been submitted to GenBank under accession no. LR595917, LR595922, OM530137 to OM530141 and OM649832 to OM649843.

## Figures and Tables

**Table 1:** In vitro susceptibility data for *C. auris* isolates against eight antifungal drugs and mutation patterns in *ERG11* and *FKS1*.

Isolate	Clinical	Minimum Inhibitory Concentration (MIC) Values (µg/mL) For							*FKS1* Sequence		*ERG11*
No.	Source	FLU	VOR	ITR	POS	AFG	MFG	AMB	HS-1	HS-2	Sequence
KW3506/17	Urine	≥128	0.25	0.5	0.063	0.031	0.016	1	WT	WT	K143R
KW3525/17	TS	≥128	0.25	0.5	0.031	0.031	0.016	1	WT	WT	K143R
KW3584/17	Urine	≥128	0.25	1	0.063	0.063	0.016	1	WT	WT	K143R
KW60/18	TS	≥128	0.5	0.5	0.031	0.063	0.016	1	WT	WT	K143R
KW87/18	Blood	≥128	0.5	0.5	0.063	0.25	0.125	1	S639T	WT	K143R
KW93/18	TS	≥128	0.5	0.5	0.031	1	1	1	S639Y	WT	K143R
KW108/18	TS	≥128	0.5	0.5	0.063	0.25	0.125	1	S639T	WT	K143R

TS, tracheal secretion; FLU, fluconazole; VOR, voriconazole; ITR, itraconazole; POS, posaconazole; AFG, anidulafungin; MFG, micafungin; AMB, amphotericin B; HS-1, hotspot-1; HS-2, hotspot-2.

**Table 2:** Non-synonymous or deletion mutations detected in hotspot-1 and hotspot-2 of *FKS1* in *C. auris* reported in different studies.

*FKS1* Hotspot Region	Codon Positions and Sequence	Specific Mutation	Micafungin MIC Range (µg/mL)	Main Reference(s)
Hotspot-1	635-FLTLSLRDP-643	ΔF635	0.5	[34]
		F635L	1	[46]
		F635Y	4 to 16	[46]
		S639F	2 to 16	[18,43,45,46,48]
		S639Y	1 to 8	[51], This study
		S639P	1 to 8	[52]
		S639T	0.125	This study
		D642Y	0.063	[18,53]
Hotspot-2	1350-DWIRRYTL-1357	R1354S	16	[46]
		R1354H	0.125	[18]

Codon positions frequently mutated in hotspot-1 and hotspot-2 of *FKS1* in the echinocandin-resistant *C. auris* are shown in bold and underlined letters.

## Data Availability

All the data are available in the published article; the datasets are available with the corresponding author and can be provided upon reasonable request.
